# Statistical measures of transcriptional diversity capture genomic heterogeneity of cancer

**DOI:** 10.1186/1471-2164-15-876

**Published:** 2014-10-08

**Authors:** Tingting Jiang, Weiwei Shi, René Natowicz, Sophia N Ononye, Vikram B Wali, Yuval Kluger, Lajos Pusztai, Christos Hatzis

**Affiliations:** Department of Internal Medicine, Yale School of Medicine, Yale Cancer Center, New Haven, Connecticut USA; Computer Science Department, Universite Paris-Est, Paris, France; Department of Pathology, Yale School of Medicine, Yale Cancer Center, New Haven, Connecticut USA

**Keywords:** Tumor diversity, Breast cancer, Basal-like subtype, Chemotherapy resistance, Dispersion distance, Hamming distance, Chemotherapy

## Abstract

**Background:**

Molecular heterogeneity of tumors suggests the presence of multiple different subclones that may limit response to targeted therapies and contribute to acquisition of drug resistance, but its quantification has remained challenging.

**Results:**

We performed simulations to evaluate statistical measures that best capture the molecular diversity within a group of tumors for either continuous (gene expression) or discrete (mutations, copy number alterations) molecular data. Dispersion based metrics in the principal component space best captured the underlying heterogeneity. To demonstrate utility of these measures, we characterized the diversity in transcriptional and genomic profiles of different breast tumor subtypes, and showed that basal-like or triple-negative breast cancers (TNBC) are significantly more heterogeneous molecularly than other subtypes. Our analysis also suggests that transcriptional diversity is a global characteristic of the tumors observed across the majority of molecular pathways. Among basal-like tumors, those that were resistant to multi-agent chemotherapy showed greater transcriptional diversity compared to chemotherapy-sensitive tumors, suggesting that potentially multiple mechanisms may be contributing to chemotherapy resistance.

**Conclusions:**

We proposed and validated measures of transcriptional and genomic diversity that can quantify the molecular diversity of tumors. We applied the new measures to genomic data from breast tumors and demonstrated that basal-like breast cancers are significantly more diverse than other breast cancers. The observation that chemo-resistant tumors are significantly more diverse molecularly than chemosensitive tumors implies that multiple resistance mechanisms may be active, thus limiting the sensitivity and accuracy of predictive markers of chemotherapy response.

**Electronic supplementary material:**

The online version of this article (doi:10.1186/1471-2164-15-876) contains supplementary material, which is available to authorized users.

## Background

Pathologists have long recognized that tumors are highly heterogeneous consisting of neoplastic cells with distinct morphological and molecular features that are associated with clinically distinct phenotypes [1]. In the past few years, large-scale next-generation sequencing provided evidence of extensive genomic diversity in histopathologically similar cancers, with each tumor effectively harboring a unique repertoire of genomic abnormalities [2–5]. Epigenetic changes that occur throughout tumorigenesis and the environmental cues conveyed through a tumor’s microenvironment contribute to the observed phenotypic heterogeneity, but genetic alterations are thought to drive the majority of tumor phenotypic variation [6].

The early large scale transcriptional profiling efforts provided the initial impetus for characterizing the phenotypic heterogeneity of cancer that allowed to discover transcriptionally-uniform cancer subtypes that were associated with distinct clinical outcomes [7, 8]. These subtypes were subsequently shown by next generation sequencing to be molecularly and genomically quite heterogeneous [9]. Although the causal drivers of intertumor heterogeneity are not clearly understood [10], genomic instability appears to be a unifying theme in many genetically diverse malignancies, acting to deregulate the control of DNA replication thus promoting proliferation and destabilizing the genome [11]. The increased clonal diversity that results from genetic instability is associated with a higher risk of progression [12], worst survival [13] and resistance to chemotherapy [14]. A recent study reported that the pre-treatment genomic diversity among cells from each of the four breast cancer subtypes is preserved after chemotherapy, but tumors with lower genetic diversity before treatment are more likely to achieve complete pathologic response (pCR), irrespective of phenotypic subtype [15].

Breast tumors of the basal-like or triple negative breast cancer (TNBC) subtype exhibit a broad range of complex structural DNA alterations [4, 16] that render them particularly challenging to treat effectively as a group with standard cytotoxic chemotherapy or with targeted therapies. Because a tumor’s transcriptional profile is an amalgam of the functional genetic and epigenetic variations that it harbors, higher intratumor genetic heterogeneity would be reflected in greater transcriptional diversity or dissimilarity between tumors of the same phenotype. Such transcriptional diversity could be the reason why developing gene-expression based predictors of chemotherapy sensitivity for basal-like cancer has been unexpectedly difficult [17, 18]. The term diversity is used here to denote the dissimilarity in gene expression or mutational profiles between individuals, which is a characteristic of a group of tumors, while the term heterogeneity here refers to the non-uniformity in genetic and cellular composition of an individual tumor.

In this paper we evaluated through extensive simulations a number of statistical metrics based on the pairwise similarity of transcriptional and genomic profiles and selected mean dispersion distance as the best measure of transcriptional diversity. Using this measure, we observed that among basal-like breast cancers, those that were chemotherapy resistant were significantly more diverse than chemotherapy sensitive cancers. These results suggest that multiple resistance mechanisms may be active among cancers of the same subtype and such heterogeneity would need to be accounted for when developing predictive models of response to chemotherapy.

## Results

### Evaluation of transcriptional diversity metrics in simulated datasets

We used two different strategies to assess transcriptional diversity among a group of profiles. In the first strategy, we considered all possible pairwise distances among the profiles the group using Pearson or cosine correlation for pairwise similarity of transcriptional profiles. In the second strategy, we used a dispersion distance metric to summarize the diversity as distance from the population centroid [19] (details in Methods). We considered either the mean or the median of the distribution of pairwise or dispersion distances as a summary of the central tendency in the group. To evaluate the performance of the different metrics, we generated artificial gene expression datasets representing scenarios that differed in the number of latent subgroups present and in the within (*σ*_*g*_) and between (*σ*_*p*_) tumor sample variance using the R package Umpire [20]. In each scenario, the log expression level for each gene was generated by a hierarchical model, in which *σ*_*g*_ controls the within-tumor variance and *σ*_*p*_ controls the variance across patients in the cohort [21].

**Figure 1:**
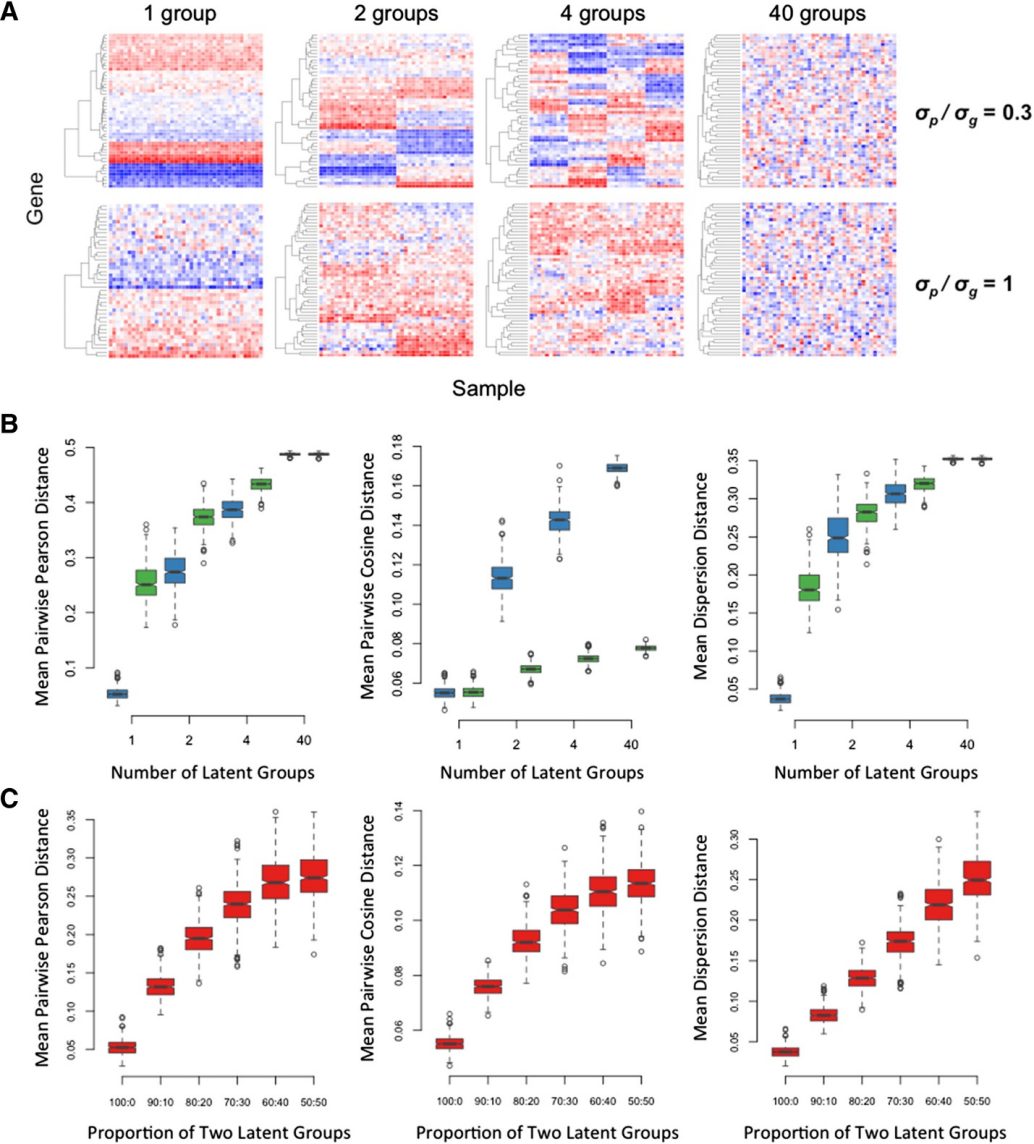
**Assessment of different transcriptional diversity metrics in simulated datasets. A)** Simulated gene expression profiles generated using a hierarchical model to independently control within sample (*σ*_*g*_) and between samples (*σ*_*p*_) transcriptional variation and the number of latent subgroups. Each profile consists of 50 genes (rows) and 40 samples (columns). Profiles for 1, 2, 4 and 40 latent subgroups are shown for low (*σ*_*p*_ */σ*_*g*_ = 0.5/1.5) and high (*σ*_*p*_ */σ*_*g*_ = 0.5/0.5) relative between-to-within sample variation. **B)** Transcriptional diversity within the simulated profiles assessed using the mean pairwise Pearson distance, the mean pairwise cosine distance, or the mean dispersion distance. The boxplots represent the distributions of these metrics obtained from 500 independent simulations of each dataset (blue: low *σ*_*p*_ */σ*_*g*_, green high *σ*_*p*_ */σ*_*g*_). **C)** Same metrics as in B assessed in a two latent subgroup dataset of 40 samples with increasing proportion of the smaller subgroup. Boxplots represent distribution over 500 independent simulations.

Figure 1A shows eight simulated scenarios (50 genes per sample, 40 samples per cohort) corresponding to 1, 2, 4, and 40 even-sized latent intra-cohort subgroups, and two levels of relative between and within-tumor variation (*σ*_*p*_*/σ*_*g*_ = 0.5/1.5 and 0.5/0.5). A smaller *σ*_*p*_*/σ*_*g*_ ratio indicates lower relative variation across tumors and therefore higher similarity in their expression profiles or lower overall diversity, whereas a larger *σ*_*p*_*/σ*_*g*_ ratio or a greater number of latent subgroups implies greater within cohort diversity. The mean of all within-group pairwise distances was calculated for each scenario repeatedly over 500 independent simulations from the same distributions. All metrics consistently increased with increasing number of latent subgroups and *σ*_*p*_*/σ*_*g*_ ratio, except for the cosine metric that exhibited lower sensitivity when the relative between and within-tumor variation was high (Figure 1B). The mean dispersion distance tracked almost linearly the increase in heterogeneity due to increasing proportion in a two latent subgroup scenario (Figure 1C). The median pair-wise distance was generally less sensitive than the mean (Additional file 1: Figure S1), and the results were robust over a broad range of genes used to compute the distances (Additional file 1: Figure S2). Based on these simulations, we selected the mean within-cohort dispersion distance as the best metric to describe intra-cohort transcriptional diversity.

### Transcriptional and genomic diversity within TCGA breast cancer subtypes

**Figure 2:**
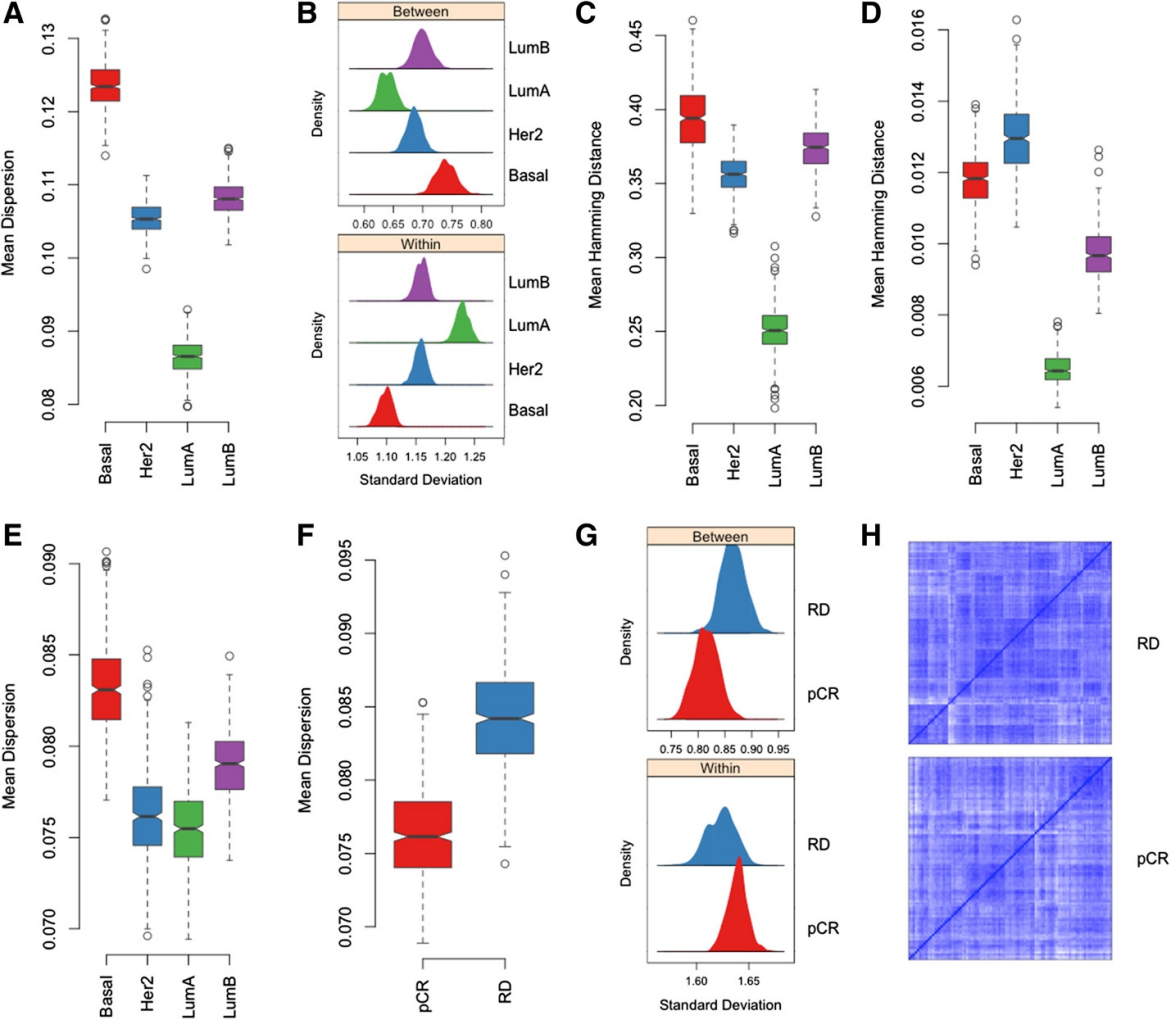
**Characterization of transcriptional and genomic heterogeneity of breast cancer subtypes. A)** Transcriptional diversity of cancers within each subtype from the TCGA gene expression data [4] captured by the mean dispersion distance metric. **B)** Distributions of within and between patient standard deviations of gene expression levels for each subtype estimated from the TCGA gene expression data. **C)** Genomic heterogeneity of DNA copy number within each subtype estimated by the mean pairwise Hamming distance in the DNA copy number profiles of cancers from the TCGA dataset. **D)** Mutational heterogeneity of subtypes estimated by the mean pairwise Hamming distance between the somatic mutation profiles of cancers from the TCGA dataset. **E)** Transcriptional diversity of cancers from the Affymetrix U133A datasets assessed using the mean dispersion distance metric. **F)** Transcriptional diversity based on mean dispersion distance of basal-like tumors that achieved pathological complete response (pCR; n = 96) or had partial or no response (RD; n = 159) to preoperative chemotherapy. **G)** Estimated distributions of within and between patient standard deviations of gene expression within the pCR and RD basal-like phenotypes. **H)** Patient-patient pairwise correlation plots clustered to show substructure within the pCR and RD basal-like tumors. The scale for the Pearson correlation coefficient ranges from 0 (white) to 1 (blue). While chemo-sensitive tumors (pCR) show less structure, resistant tumors (RD) show a greater number of subgroups with relatively uniform gene expression. Boxplots represent the distribution of the corresponding metric obtained from 500 bootstrap resampling iterations of 100 cases from each subtype or subgroup.

We assessed the transcriptional heterogeneity within the four breast cancer subtypes as assigned by the PAM50 classifier using gene expression data from The Cancer Genome Atlas (TCGA) breast cancer project (N = 547; Table 1) [4]. Gene expression data were used as provided and probe sets with low expression or low variation across the dataset were filtered as described in the Methods. Mean dispersion distance was computed in groups of 100 cases from each subtype, repeatedly sampled with replacement over 500 bootstrap iterations. Overall, tumors of the luminal A subtype were significantly less heterogeneous than other subtypes (permutation test *P* < 10^-5^), while basal-like tumors had the highest transcriptionally diversity (permutation test *P* < 10^-5^) (Figure 2A). Estimation of within-patient and between-patient variance (see Methods) showed that luminal A tumors had the highest within-patient gene expression variance but the lowest between-patient variance (smaller *σ*_*p*_*/σ*_*g*_ ratio), whereas the reverse was true for basal-like tumors that show a relatively narrow range of expression within each tumor but more diverse expression across tumors (Figure 2B). Comparing the luminal subtypes, luminal B tumors appear transcriptionally significantly more diverse (permutation test *P* < 10^-5^), exhibiting a higher estimated *σ*_*p*_*/σ*_*g*_ ratio, similar that of HER2-enriched tumors (Figure 2A, B).

**Table 1:** **Breast cancer TCGA datasets used in this study**

Data	TCGA file link	N
Tumor Information	https://tcga-data.nci.nih.gov/docs/publications/brca_2012/BRCA.datafreeze.20120227.txt	466
PAM50 Subtype	https://tcga-data.nci.nih.gov/docs/publications/brca_2012/BRCA.547.PAM50.SigClust.Subtypes.txt	466
Gene Expression-Level 3	http://tcga-data.nci.nih.gov/docs/publications/brca_2012/BRCA.Gene_Expression.Level_3.tar	547
Somatic Mutations	http://tcga-data.nci.nih.gov/docs/publications/brca_2012/genome.wustl.edu_BRCA.IlluminaGA_DNASeq.Level_2.3.2.0.tar.gz	463
Copy Number Alterations	http://tcga-data.nci.nih.gov/docs/publications/brca_2012/brca_hg19_qc.merged.seg	466
Gene Models (RefSeq)	UCSC table browser with track “RefSeq Genes” of genome version Feb. 2009 GRCh37/hg19 (http://genome.ucsc.edu/cgi-bin/hgTables)

To examine whether the higher transcriptional diversity observed in basal-like tumors is associated with greater genetic heterogeneity, we evaluated the diversity in somatic mutation profiles and DNA copy number variation as categorical data using the mean pairwise Hamming distance [22] to assess intra-cohort diversity of genomic profiles (see Methods). The mean pairwise Hamming distance was able to track the population diversity in the different simulation scenarios (Additional file 1: Figure S3). As observed at the transcriptome level, basal-like cancers exhibited the highest diversity in the distribution of copy number aberrations and the second highest mutational heterogeneity, whereas luminal A tumors were genomically the least diverse (Figures 2C, 2D). Our results are consistent with the assessment in the original TCGA publication that reported the highest overall rate of genome alteration in basal-like tumors and the lowest in luminal A tumors [4]. Moreover, the almost indistinguishable patterns of molecular diversity that we observed in the genomic and transcriptomic profiles of breast tumors suggest that a measure of the transcriptional diversity could be a effective surrogate measure of the underlying genomic heterogeneity.

### Validation of transcriptional diversity patterns within breast cancer subtypes using microarray datasets

We evaluated the generalizability of the results obtained on the TCGA data using publicly available gene expression datasets (Table 2) to assess the transcriptional diversity of basal-like (n = 403), HER2-enriched (n = 171), luminal A (n = 515) and luminal B (n = 210) breast cancers. We calculated the mean dispersion distance of transcriptional profiles within each subtype over 500 bootstrap iterations in groups of 100 cases per subtype. The pattern of transcriptional diversity was very similar to that observed in the TCGA dataset (Figure 2E), with basal-like cancers being the most diverse and luminal A cancers being the most uniform (permutation test *P* < 10^-5^), except that HER2-enriched cancers in this cohort appeared less diverse.

**Table 2:** **Breast cancer Affymetrix U133A microarray datasets**

GEO dataset	N	PAM50 subtype					Response group basal-like	
		Basal	Her2	LumA	LumB	Normal	pCR	RD
GSE11121	200	23	18	110	24	25	0	0
GSE20194	91	18	30	16	13	14	7	6
GSE20271	81	19	15	22	9	16	4	15
GSE2034	286	63	35	99	51	38	0	0
GSE22093	96	44	14	16	12	10	18	24
GSE25055	310	120	22	97	46	25	45	73
GSE25065	198	68	15	62	34	19	22	41
GSE7390	198	48	22	93	21	14	0	0
*Total*	1460	403	171	515	210	161	96	159

### Basal-like breast cancers that respond to chemotherapy are transcriptionally homogeneous

Among the 403 basal-like cases in the Affymetrix dataset, 255 cases received preoperative combination chemotherapy (paclitaxel, 5-fluoracil, cyclophosphamide and doxorubicin), which allowed us to compare the transcriptional diversity of exceptionally chemotherapy sensitive cancers that achieved pathological complete response (pCR, n = 96) to those who had partial or no response as evidenced by viable amounts or residual cancer after preoperative chemotherapy (RD, n = 159)(Table 2). We compared the transcriptional diversity of cancers from the two response groups by calculating the mean dispersion distance in sets of 100 cases per group over 500 bootstrap iterations. Chemotherapy resistant basal-like cancers showed significantly greater transcriptional diversity compared to chemotherapy sensitive cancers (permutation test *P* < 10^-5^; Figure 2 F). These resistant cancers had narrower within tumor expression range but a greater variation between cancers (Figure 2G), and also a greater number of latent transcriptionally homogeneous subtypes (Figure 2H). Our results provide evidence that pathological complete response is associated with significantly lower pretreatment transcriptional diversity, corroborating previous reports of a significantly lower genetic divergence in tumors achieving pCR [15].

### Transcriptional diversity extends at the level of individual pathways

The varying degree of transcriptional diversity within different breast cancer subtypes raises the question whether the observed heterogeneity is restricted to only genes from a few pathways or whether it is more of a global transcriptional phenomenon. We defined 50 pathways from the Kyoto Encyclopedia of Genes and Genomes (KEGG) [23] that represent all biological processes but have minimal overlap in gene membership. The number of genes in these pathways ranged from 11 (folate biosynthesis) to 389 (olfactory transduction) (see Methods).

**Figure 3:**
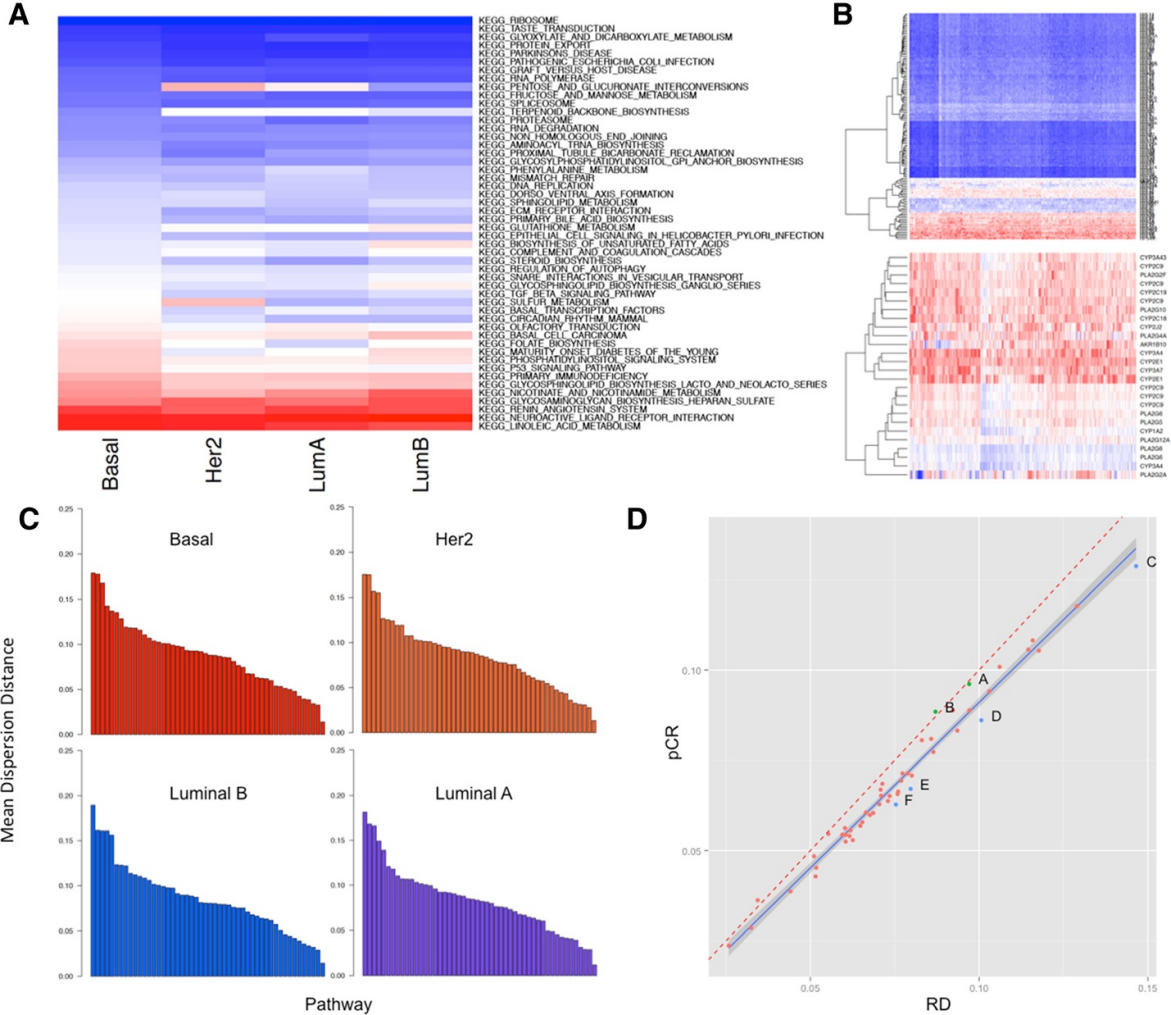
**Transcriptional diversity of 50 KEGG biological pathways within breast cancer subtypes from the Affymetrix dataset. A)** Heatmap of mean dispersion distance within breast cancer subtypes considering genes in each of the 50 KEGG pathways. Pathways (rows) are ranked from the least diverse at the top to the most diverse at the bottom. Blue represents low and red high mean dispersion. **B)** Detailed expression heat maps for basal-like cancers showing the heterogeneity of gene expression for genes in the least heterogeneous (ribosome metabolism; top) and the most heterogeneous pathways (linoleic acid metabolism; bottom). Blue represents low and red high expression level. **C)** Distribution of pathway-based transcriptional diversity within each subtype. Pathway-level mean dispersion distances were calculated by bootstrap as described in Supplementary Methods. **D)** Comparison of pathway-level transcriptional diversity between two clinically distinct phenotypes of basal-like cancers, an extremely chemosensitive (pCR) and a chemoresistant (RD). Points on the plot represent the average pathway-level mean pairwise dispersion obtained from boostrap within each of the 50 pathways. The dashed red line is the diagonal, indicating equal transcriptional diversity between the two response phenotypes. The regression line (blue solid line) its 95% pointwise confidence interval (grey area) is consistently below the diagonal suggesting greater transcriptional diversity for RD cancers throughout the 50 pathways. Pathways that were extreme outliers from the trend described by the regression line were identified by quantile-quantile plots of the standardized residuals. These pathways are indicated with letters as following: A – sphingolipid meta, B – SNARE interactions in vesicular transport, C – basal cell carcinoma, D – dorso-ventral axis formation, E – non-homologous end joining (DNA repair), F – folate biosynthesis.

We applied the same bootstrapping procedure to calculate the mean dispersion distance within breast cancer subtypes, but now using only genes from each of the 50 pathways. Most pathways showed the same order of diversity across the four subtypes as that observed globally, suggesting that transcriptional diversity is a global characteristic of the tumors and not primarily driven by heterogeneity in a few biological processes (Figure 3A, C). Certain pathways showed remarkably low diversity in all subtypes indicating a tight transcriptional coordination of genes involved in biological processes that are vital for all cells (e.g. ribosome metabolism, protein export, glyoxylate/dicarboxylate metabolism; Figure 3A, B). Only a few pathways were distinctively diverse within each subtype (Figure 3C), and these were the same in all breast cancer subtypes (linoleic acid metabolism, renin/angiotensin system, and neuroactive ligand/receptor interaction) (Figure 3A, B).

We also compared the pathway-level transcriptional diversity of chemotherapy sensitive (pCR) or resistant (RD) basal-like cancers. Almost all pathways (48/50) were transcriptionally more diverse in the chemotherapy resistant group (Figure 3D), suggesting that the greater transcriptional diversity in these tumors is global and not restricted to certain pathways (slope of regression line = 0.917, 95% confidence interval 0.877 to 0.956). Pathways with greater diversity in RD cancers relative to pCR cancers included basal-cell-carcinoma, which includes genes from the Hedgehog, Wnt, TGF and p53 signaling pathways, folate biosynthesis, and dorso-ventral axis formation pathway involving genes from several key signaling pathways including the MAPK and NOTCH (Figure 3D).

## Discussion

In this study, we showed that the mean dispersion and the mean pairwise Hamming distance provide quantitative metrics that capture the transcriptional and genomic diversity of breast cancer subtypes. We demonstrated that each subtype is characterized by a different degree of transcriptional and genomic diversity and provided statistical evidence that basal-like breast cancers are the most and luminal A cancers are the least heterogeneous. While this has been suggested in the literature [4, 16, 24] it has never been previously demonstrated in statistical terms. The difference in transcriptional diversity between subtypes appears to concern the entire transcriptome with the exception of a few biological processes that are critical for cell survival, suggesting that basal-like and luminal A cancers differ in fundamental ways in how tightly gene co-expression is regulated within cells. These results are also consistent with greater intratumor cellular heterogeneity in basal-like cancers due to coexistence of either genomically different clonal populations or genomically similar cells exhibiting greater diversity in cellular states. But since we also observed identical trends in the relative heterogeneity of DNA copy number alterations and somatic mutations between the subtypes, the results strongly suggest that genomic alterations leave a major imprint on transcriptional profiles [25] and likely drive the higher transcriptomic diversity of basal-like cancers. Transcriptional diversity can thus be considered as a surrogate measure of the underlying genomic heterogeneity of cancers. To further elaborate, we would expect repeat biopsies from a tumor with high intratumor heterogeneity to show greater transcriptional diversity, as biopsies may consist of different mixtures of clonal cells. High intratumor clonal variation would also imply higher variation between two such tumors and therefore a greater transcriptional diversity.

We also demonstrated that two clinically very distinct subtypes of basal-like cancers, chemotherapy resistant and highly chemotherapy sensitive cancers, which cannot not be reliably separated using gene expression based multivariate prediction models [17, 18], show significant differences in transcriptional diversity (permutation test *P* < 10^-5^). Chemotherapy resistant cancers had much greater transcriptional diversity across most biological processes. Greater diversity in regulatory and metabolic pathways could confer greater resilience against cytotoxic insults [26]. Pathways that are expressed more diversely in chemotherapy resistant basal-like cancers may provide biological insights into the mechanisms of chemotherapy resistance.

Finally, our results generate new insights as to why it may be difficult to predict chemotherapy response in basal-like cancers. Due to the greater transcriptional diversity in chemotherapy resistant tumors, a single gene expression signature of resistance would fail to represent adequately the spectrum of resistance mechanisms present and thus limit the sensitivity and accuracy of such predictors. This has been demonstrated to be the case recently through insightful simulations [27]. Using genomic DNA copy number alterations to substratify basal-like cancers into genomically more uniform groups could perhaps improve predictability, but this strategy will require considerably larger cohorts. Alternatively, using a single metric that captures the within-patient molecular heterogeneity may prove a more effective strategy to predict general chemotherapy sensitivity of a given cancer.

## Conclusion

We presented and systematically evaluated a quantitative measure to capture transcriptional and genomic diversity among cancers. Results from different types of genomic data consistently demonstrated that basal-like cancers are the most heterogeneous while luminal A the least. Such diversity appears to be a global characteristic reflected in most biological process. Finally, we also showed that greater transcriptional diversity is also associated with basal-like cancers that are resistant to chemotherapy, suggesting that molecular heterogeneity is a manifestation, potentially a causal one, of treatment resistance. This also explains the difficulty in developing generalizable gene signatures that are predictive of chemotherapy response in the highly heterogeneous triple-negative breast cancers.

## Methods

### Breast cancer datasets and processing

#### The Cancer Genome Atlas (TCGA) Dataset

Datasets were downloaded from the TCGA breast carcinoma web site (https://tcga-data.nci.nih.gov/docs/publications/brca_2012/) [4] (Table 1). Gene expression data by Agilent 244 K arrays were available for 547 cases. DNA copy number alteration data by Affymetrix 6.0 SNP arrays were available for 466 cases. Somatic mutation data by whole exome sequencing were available on 463 cases. The TCGA PAM50 subtype classification was downloaded also from the same site. A sample list is provided in Additional file 2: (TCGA sample ID.xlsx).

#### Affymetrix U133A dataset

A total of 1460 breast cancer profiles from eight breast cancer datasets that were uniformly profiled on the Affymetrix U133A microarray were obtained form the Gene Expression Omnibus (http://www.ncbi.nlm.nih.gov/geo/). Because of partial overlap in several of these datasets, the selected unique samples from each GEO dataset are provided in Additional file 3: (Affy_sample_info1460.txt). A summary of the combined data set is given in Table 2.

#### Data preprocessing

The TCGA gene expression data were used as provided without any additional processing. The segmented log2 DNA copy number values were trichotomized as deletion (score < -0.3), amplification (score > 0.3) or no effect. We aggregated the segment-level alterations at the gene level using RefSeq gene models. A gene was labeled as deleted or amplified if the entire transcribed region was in a deleted or amplified segment. Of the 45,918 RefSeq genes, 22,271 were amplified or deleted in at least one of the patients in the cohort and were used in the calculation of the Hamming distance. The somatic mutation data were provided at the gene level in the TCGA dataset (any mutation vs no mutations) and did not require any further processing.

The Affymetrix U133A data files were compiled in a single dataset, normalized by MAS5, scaled to a target intensity of 600 and log2 transformed. Principal component analysis (PCA) was applied to detect potential batch effects across the datasets. We used the empirical Bayes framework procedure ComBat [28] as implemented in the R package sva [29] to remove batch effects due to different datasets. We used PCA after batch effect removal to verify lack of batch effects after correction (Additional file 1: Figure S4). A previously validated PAM50 algorithm for Affymetrix-based expression data was used to generate breast cancer subtype classification for each cancer in the dataset [30]. Table 2 provides a summary of all the datasets used in this study and the subtype distribution within each dataset. The 161 normal-like cases were excluded from the analysis. Prior to analysis, the combined, batch-adjusted dataset was filtered to remove probe sets in the lowest expression quartile and those in the lowest median absolute deviation (MAD) decile. The remaining 14,505 probe sets were used in subsequent analyses.

### Collection and processing of KEGG pathways

The 186 pathways used in this study were collected from the Molecular Signatures Database [31] (MSigDB; http://www.broadinstitute.org/gsea/msigdb/index.jsp) and correspond to gene sets derived from the KEGG pathway database [23] (http://www.genome.jp/kegg/pathway.html). We processed the original list of 186 pathways to produce a reduced set of pathways that had minimal overlap in gene membership. We broadly followed the steps described previously [32]. First, we calculated a matrix of pairwise pathway similarities derived from the hypergeometric distribution as follows. If *N*, *N*_*A*_ and *N*_*B*_ represent the genes in all pathways, in pathway A and in pathway B respectively, and these two pathways have *n* genes in common, then the degree of enrichment of pathway B in genes also included in pathway A is given by the hypergeometric distribution h(*N, N*_*A*_*, N*_*B*_) [33]. This can be visualized as a 2x2 contingency table cross-tabulating the number of genes in the two pathways (pathway A vs not pathway A by pathway B vs not pathway B). The objective is to test whether genes in pathway B are over-represented among genes in pathway A compared to genes not in pathway A. Thus a rejection of the null hypothesis of independence between the two dimensions of the 2x2 table is evidence for significant over-representation or overlap between the two pathways. If we let *X* denote the number of genes in common, then the probability of two unrelated pathways (under the null hypothesis) having at least *n* genes in common is given by


Pathways for which the null hypothesis was rejected at the 0.05 level, i.e *P*(*X* ≥ *n*) < 0.05 were labeled as similar (value of 1) and the remaining as dissimilar (value of 0) in the pathway similarity matrix. Pathways with the highest degree of overlap were identified as those with the highest row or column sum in the similarity matrix and were removed recursively by removing both the corresponding column and the row from the pathway similarity matrix. The resulting 50 pathways consisted of almost disjoint genes sets and were used in subsequent analyses.

### Distance measures

#### Pearson distance

If the pairwise Pearson’s correlation coefficient between two expression vectors (profiles) is denoted by *ρ,* the Pearson’s pairwise distance was calculated as


All the unique pairwise distances within a set of profiles are computed and the distribution of pairwise distances is summarized by its mean or its median.

#### Cosine distance

Given expression vectors *X*_*i*_*, X*_*j*_, their cosine distance is given by


where ∥*X*∥ represents the L2-norm of vector *X.* The common definition of the cosine similarity measure is the cosine of the angle between two vectors. Here, we define the cosine dissimilarity or distance measure as the normalized angle between the two vectors. The above two dissimilarity measures are related, since the Pearson’s correlation is essentially equivalent to a centered cosine similarity measure:


As above, all the unique pairwise distances within a set of profiles are computed and the distribution of pairwise distances is summarized by its mean or its median.

#### Dispersion distance

For the calculation of dispersion distances, the distance matrix *A* of all pairwise Pearson distances is centered by subtracting the row and column means and then adding the overall mean to each element of *A*. The spectral decomposition of the resulting centered matrix defines the principal coordinates. Vectors are mapped to the full principal coordinate space and their Euclidian distance from the overall centroid in this space defines the distance or dissimilarity from each vector to its centroid [34]. The overall dispersion distance for a set of vectors is then calculated as the mean or median dispersion over all vectors. Computations were performed using the R language package vegan [35].

#### Hamming distance

For categorical vectors we used the pairwise Hamming distance for character strings (not ordinal) to assess dissimilarity [22]. Specifically, for categorical vectors *X*_*i*_*, X*_*j*_ of length *L*,


where *δ*(*a*, *b*) = 1 when *a* is the same as *b* and zero otherwise. The overall Hamming dispersion distance for the set of categorical profiles was computed as the mean or median pairwise Hamming distance.

#### Permutation test for assessing significance in difference of mean distances

We used a permutation procedure to compare the bootstrap estimates of the mean group distances between two groups of samples. Two vectors, each containing 500 bootstrap estimates of the mean distance within each group, were concatenated to form a 1000 element vector. This vector was resampled without replacement 10^5^ times and for each permutation the average of the first 500 elements was compared to the average of the last 500 elements. This difference was then compared to the original difference in the mean distances between the two groups. The p-value, which represents the probability of observing by chance a difference between the bootstrapped mean distances at least as high as the observed, was then computed as (1 + #(D > d))/(1 + R), where D is the difference in means from the permuted vectors, d is the difference in means from the original vector, and R = 10^5^ is the number of random permutations.

### Generation of simulated datasets

We used the Umpire R language package to simulate realistic gene expression datasets with controlled within and between patient variation in gene expression [20]. In each simulated scenario we considered 50 genes per case and 40 cases. We assumed that all the genes are expressed in all patients and that the expression of each gene across patients follows a log-normal distribution with parameters *μ*_*g*_ and *σ*_*P*_, i.e. log(*X*_*i*_) ∼ *Normal*(*μ*_*g*_, *σ*_*P*_). The mean log expression *μ*_*g*_ is assumed to have a normal distribution across genes with mean *μ*_0_ and standard deviation *σ*_*g*_, i.e. *μ*_*g*_ ∼ *Normal*(*μ*_0_, *σ*_*g*_). It was further assumed that the between-sample gene expression variance *σ*_*P*_ follows an inverse Gamma distribution with hyperparameters *α* = 15 and *β* = 7, which correspond to a mean *σ*_*P*_ equal to 0.5. The mean *μ*_0_ was set equal to 8 for all simulations. In the first scenario shown in Figure 1A, the within sample standard deviation *σ*_*g*_ was set to 1.5, whereas in the second scenario it was set to 0.5. Samples from different latent groups were generated by resampling with the same set of hyperparameters. Additional file 1: Figure S5 shows the effect of the various parameters of the hierarchical model on the estimated diversity of the resulting set of gene expression profiles. Binarized profiles were simulated in the same way and then thresholded using the function *Z* > 1.5, where *Z* = (*X* – *M*)/*SD*, *X* is the continuous gene expression value and *M*, *SD* are the gene-wide and sample-wide (overall) mean and standard deviation of expression.

### Estimation of gene expression statistics from profiles

If *X*_*ij*_ is the expression of gene *i* in sample *j* then *m*_*i*_ = *E*(*X*_*ij*_) is the centroid of gene expression levels, which is also expressed more compactly as **m** = *E*_*P*_(**X**). The mean of the centroid vector is the overall mean expression or *μ*_0_ = *E*_*g*_(**m**) = *E*_*g*_(*E*_*P*_(**X**)). The within sample variance is estimated from the variance of the centroid vector as . Finally, the between sample variance is estimated from the mean of the variance vector **s**^2^ = *V*_*P*_(**X**), or .

Additional file 1: Figure S6 shows the parameters estimated from the simulated scenarios shown in Figure 1A. The between sample standard deviation (SD) was set to 0.5 in all these scenarios. As shown in Additional file 1: Figure S6A, the between sample SD estimate is nearly unbiased when there is one latent group, with the variance of the estimate being very small in the low between-to-within SD scenario (S) but considerably larger in the high between-to-within SD scenario (U). Furthermore, an increasing number of latent groups results in greater between sample SD, reflecting the greater population diversity, but the effect is more moderate in the high between-to-within SD scenario (U). Similarly, the estimate of the mean within sample SD is nearly unbiased (1.5 for S and 0.5 for U) when there is one latent group, but an increasing number of latent groups reduces the estimate of the within sample SD. Therefore, a greater number of latent groups in a cohort will be bias upwards the estimated between sample variance and bias downwards the estimated within sample variance.

### Scripts/Code

All the R scripts required to generate the datasets and run the analyses presented in this manuscript are provided in Additional file 4.

## Electronic supplementary material

Additional file 1: **Supplementary Figures.** (PDF 4 MB)

Additional file 2: **TCGA_sample ID.txt: tab-delimited text file listing the sample, PAM50-subtype classification, and whether gene expression, somatic mutation or copy number variation data was available for each sample.** (TXT 14 KB)

Additional file 3: **Affy_sample_info1460.txt:** **tab-delimited** **text file listing the GEO sample id, GEO dataset series id, the response** **category (pCR/RD) or NA if not available, and the** **PAM50-subtype** **classification.** (TXT 39 KB)

Additional file 4: **Jiang_RScripts.zip: Archive containing R scripts for the analyses presented in this manuscript. Scripts included:** **supply-code-simulation.** R Code for generating simulated expression datasets and heterogeneity calculation. supply-code-TCGA.R Code for TCGA data import, heterogeneity calculation and comparisons. supply-code-het-affy.R Code for Affymetrix data normalization, heterogeneity calculation and comparisons. KEGG_Path_NonOverlap.R Code for generation of 50 KEGG pathways with minimum degree of overlap in gene membership. File “186KEGG.Rdata” with KEGG pathway gene lists needed by this script is also provided. (ZIP 57 KB)

## Abbreviations

TNBC: Triple-negative breast cancer
TCGA: The Cancer Genome Atlas
PAM50: Minimal gene set for classifying intrinsic subtypes for breast cancer
pCR: Pathologic complete response
RD: Residual disease
PCA: Principle component analysis
MAD: Mean absolute deviation
KEGG: Kyoto Encyclopedia of Genes and Genomes.
