# Supplementary material for: Statistical measures of transcriptional diversity capture genomic heterogeneity of cancer
Source: BMC Genomics. 2014 Oct 8;15(1):876. doi: 10.1186/1471-2164-15-876 (PMC4197225; doi:10.1186/1471-2164-15-876)
Supplement: Supplementary file 1 — Additional file 1: Supplementary Figures. (PDF 4 MB) [file 12864_2014_6541_MOESM1_ESM.pdf]

Supplementary Figures

Statistical Measures of Transcriptional Diversity Capture Genomic Heterogeneity of Cancer

Tingting Jiang[1], Weiwei Shi[1], René Natowicz[2], Sophia N. Ononye[1], Vikram B. Wali[1], Yuval Kluger[3], Lajos Pusztai[1], Christos Hatzis[1]

Departments of [1]Medicine and [3]Pathology, Yale University School of Medicine, Yale Cancer Center, New Haven, Connecticut, USA
[2]Universite Paris-Est, France
Correspondence should be addressed to CH (christos.hatzis@yale.edu)

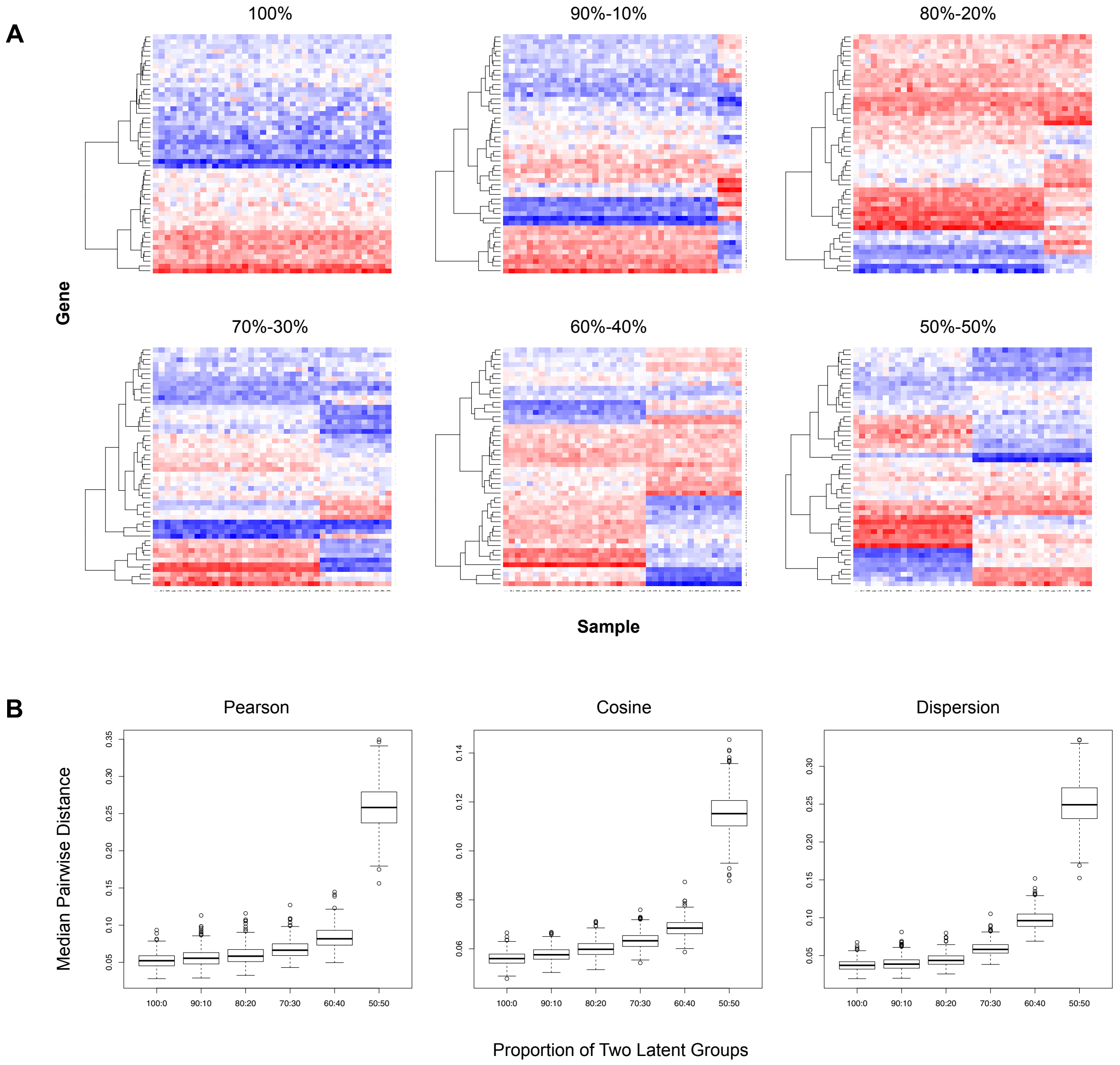

Figure S1. Assessment of transcriptional diversity metrics on simulated datasets. A) Simulated gene expression profiles generated using a hierarchical model to independently control within sample (σ_g) and between samples (σ_p) transcriptional variation and the number of latent subgroups. Each profile consisted of 50 genes (rows) and 40 samples (columns). Two latent groups were simulated form the same distribution with σ_g/σ_p = 0.5/1.5 with the proportion of cases from each latent group ranging from 100-0 to 50-50. B) Transcriptional diversity of the simulated profiles assessed using the median pairwise Pearson or cosine distances or the median dispersion, as indicted on the plots. The median metrics are less sensitive than the corresponding mean metrics (Figure 1C).

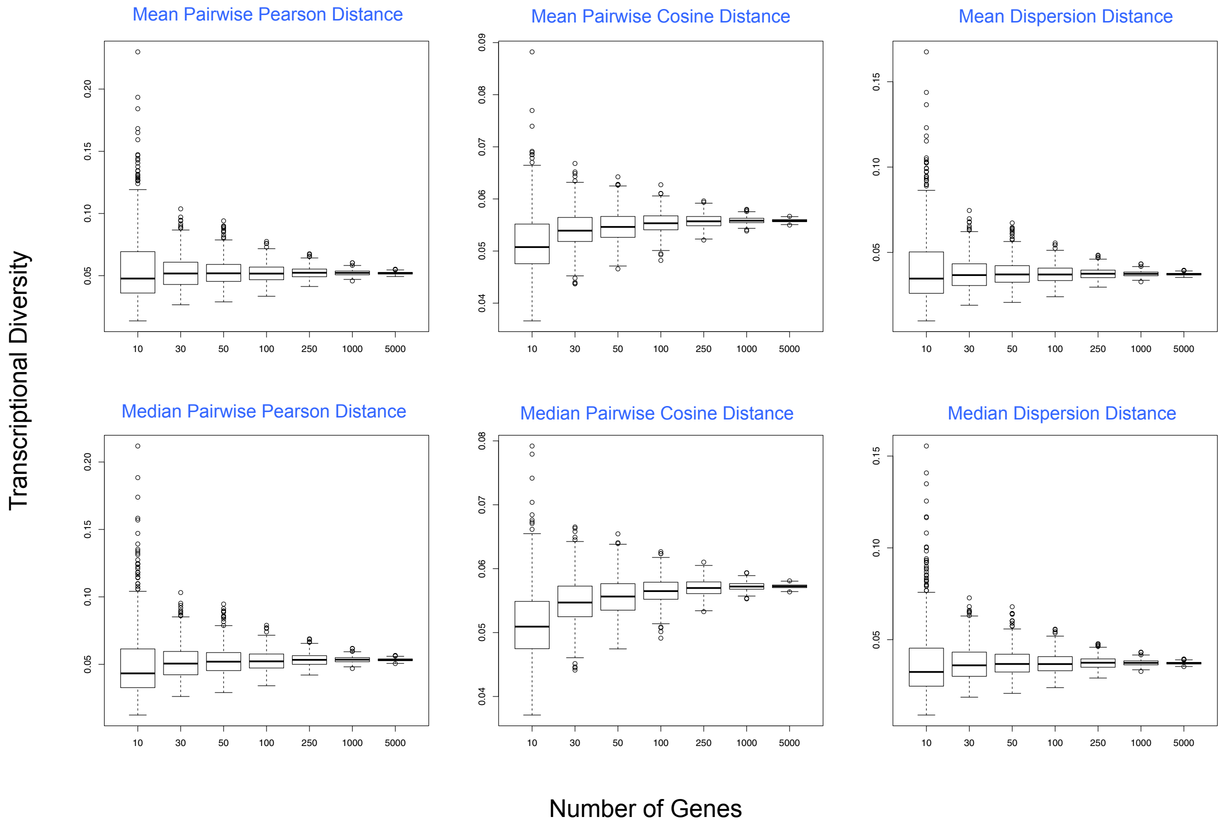

Figure S2. Effect of the number of genes used in the calculation of pairwise transcriptional distances. Expression profiles were simulated as described in the Supplementary Methods from a single latent group with a within-to-between standard deviation ratio of σ_g/σ_p = 0.5/1.5. In each case profiles including 10 to 5000 genes were generated for 50 independent cases, and each scenario was repeated 500 times. The plots show the summaries over these 500 simulations for the three different metrics (pairwise Pearson distance, pairwise cosine distance and dispersion distance) summarized either by the mean (top row) or median (bottom row) of the distribution. Calculating transcriptional diversities over smaller signatures (e.g. pathways) does not appear to introduce significant bias, but it does increase the variance of the estimate.

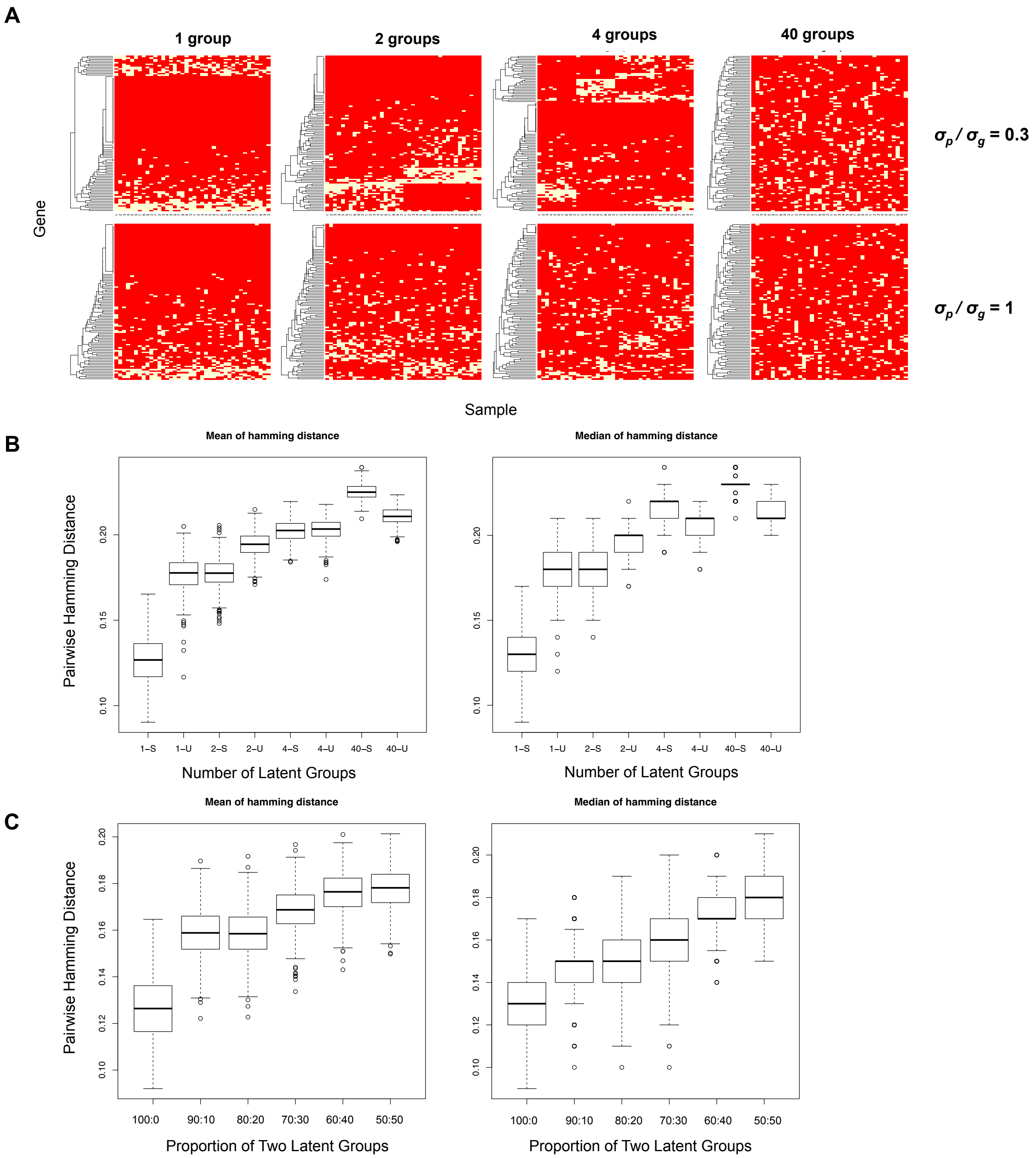

Figure S3. Assessment of mutational diversity metrics on simulated datasets. A) Simulated sparse mutational profiles generated from the same hierarchical models as the gene expression data. Each continuous expression profiles included 100 features and 40 patients and was generated as described before. The continuous profiles were subsequently binarized using the threshold function Z > 1.5, where Z = (X – M)/SD, X is the gene expression value and M, SD are the gene-wide, profile-wide mean and standard deviation of expression. B) Mutational

diversity estimated from the simulated datasets in A using mean or median pairwise Hamming distance as the diversity metric. C) Mutational diversity in simulated 2-latent class sparse binary datasets with increasing proportion of the smaller subgroup.

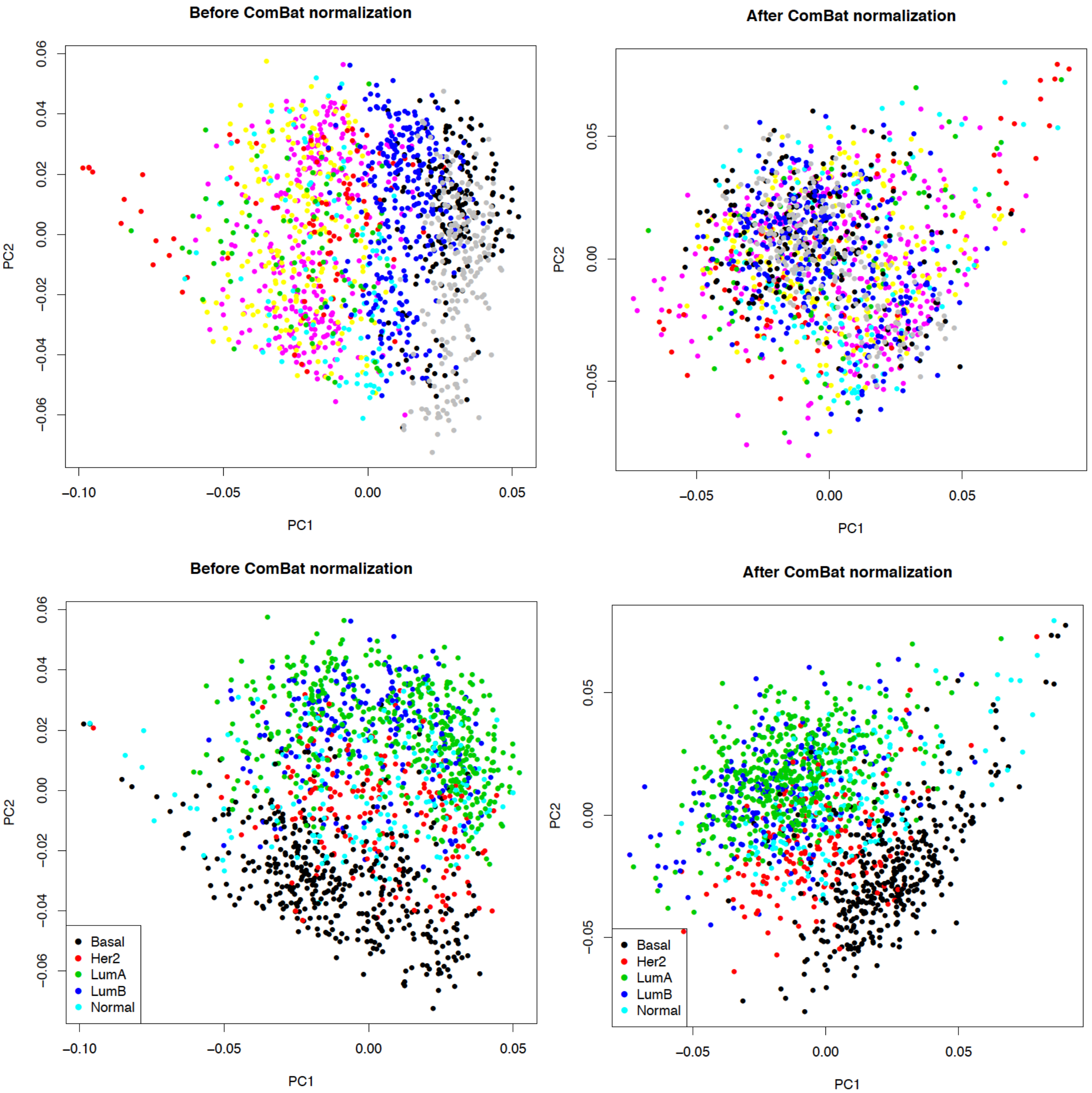

Figure S4. Scatterplots of first two principal components for the 8 microarray datasets before and after empirical Bayes (ComBat) batch effect removal. Top: datasets are indicated by different colors. Bottom: PAM50 subtypes are indicated by different colors. ComBat successfully removes dataset-related batch effects (top), while preserving the differences between subtypes (bottom).

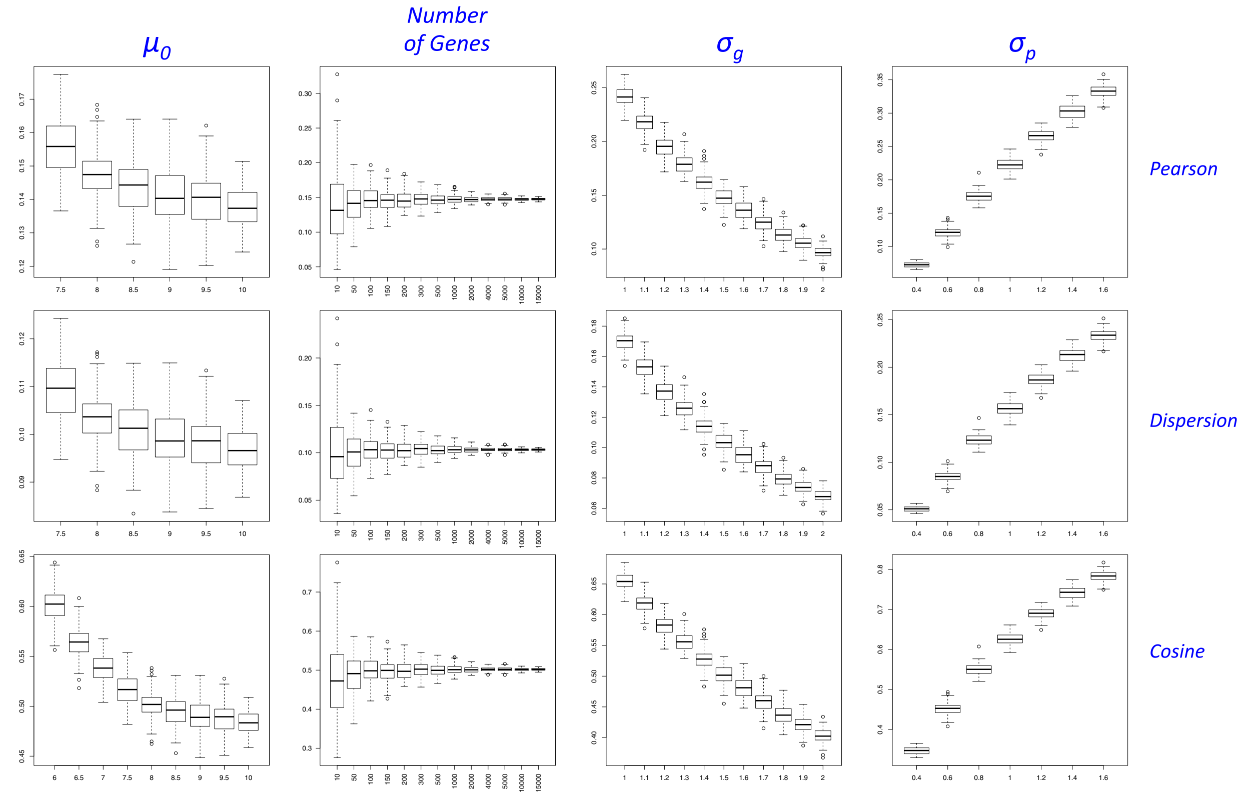

Figure S5. Estimation of transcriptional diversity on gene expression data generated by the hierarchical Umpire model using different parameters. Distributions represent results from 500 independent realizations for each set of parameters. Overall, the number of genes in the profile does not affect the estimated diversity. The mean expression level μ_0 appears to have a moderate effect on diversity, with increasing mean resulting in reduced diversity. This is expected as this results in lower within-sample coefficient of variation of expression levels. Increasing within-sample standard deviation σ_g provides a broader range of expression levels within each sample and thus stronger "banding" or structure, thus reducing the overall diversity. Finally, increasing the between-sample standard deviation of gene expression introduces considerably greater diversity between individuals, which is clearly captured by the diversity metrics.

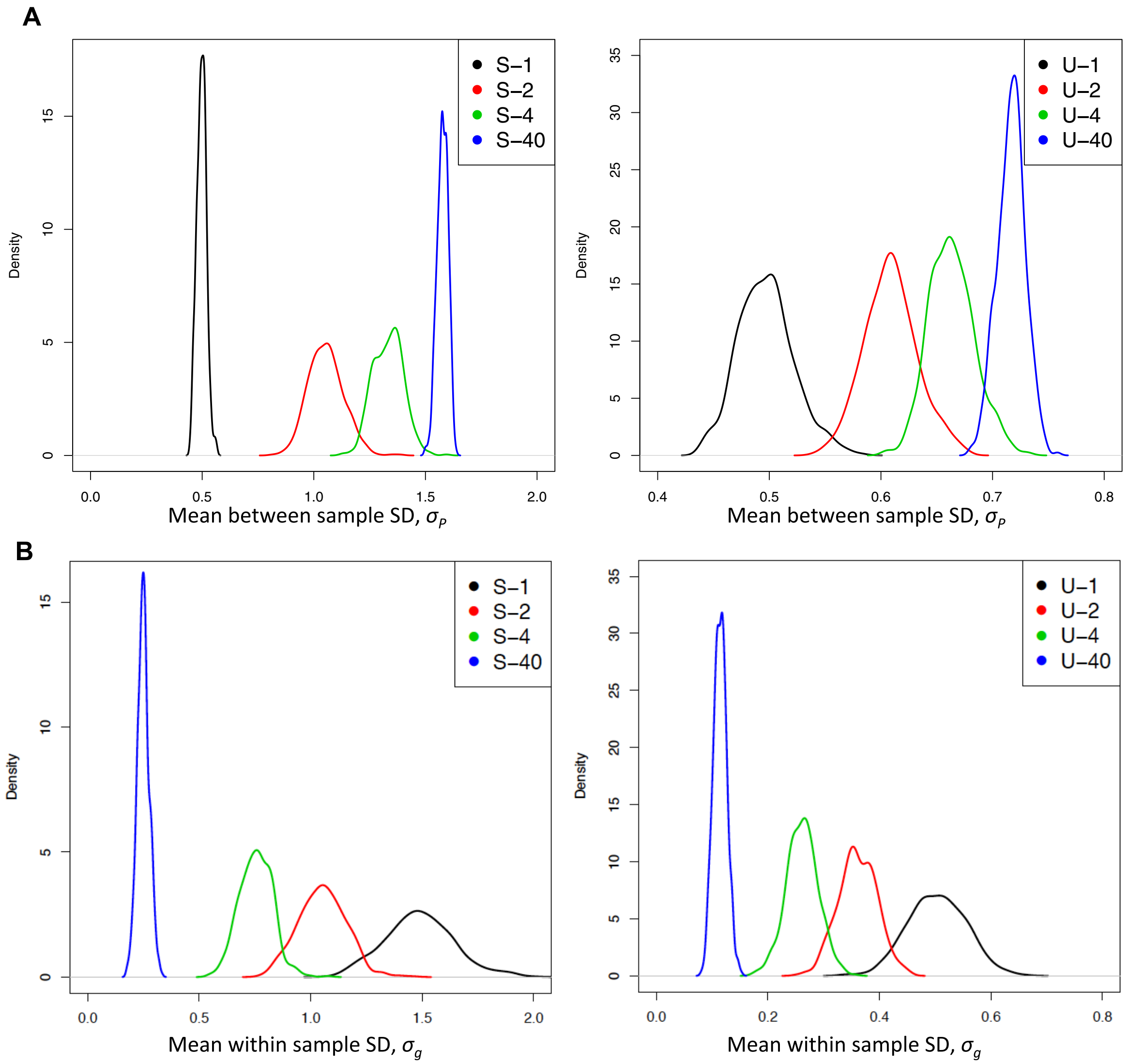

Figure S6. Between (A) and within (B) sample standard deviations (SD) estimated from 500 independent simulations of the scenarios shown in Figure 1A. Two scenarios are shown in the two columns that correspond to the two different between-to-within SD ratios depicted in Figure 1A: low ratio ($\sigma_P \ / \ \sigma_g = 0.3$) or structured (S) and high ratio ($\sigma_P \ / \ \sigma_g = 1$) or unstructured (U). Scenarios with different number of latent groups are shown with different colors.
